# Continuation of atezolizumab plus bevacizumab beyond initial progressive disease: clinical benefits in patients with unresectable hepatocellular carcinoma – a multicenter cohort study

**DOI:** 10.3389/fimmu.2025.1653456

**Published:** 2025-09-11

**Authors:** Takaya Tabuchi, Nobuhito Taniki, Keisuke Ojiro, Ryosuke Kasuga, Yukie Nakadai, Po-Sung Chu, Shingo Usui, Shunsuke Shiba, Toshiyuki Tahara, Hirokazu Komatsu, Yuriko Fujita, Fumihiko Kaneko, Hitomi Hoshi, Akihiro Yamaguchi, Seiichiro Fukuhara, Yukishige Okamura, Hideaki Kanamori, Hirotoshi Ebinuma, Masashi Tamura, Jitsuro Tsukada, Yasushi Hasegawa, Yuta Abe, Minoru Kitago, Masahiro Jinzaki, Yuko Kitagawa, Takanori Kanai, Nobuhiro Nakamoto

**Affiliations:** ^1^ Division of Gastroenterology and Hepatology, Department of Internal Medicine, Keio University School of Medicine, Tokyo, Japan; ^2^ Department of Gastroenterology, Ichikawa General Hospital, Tokyo Dental College, Chiba, Japan; ^3^ Department of Gastroenterology, Saiseikai Utsunomiya Hospital, Tochigi, Japan; ^4^ Department of Gastroenterology, Yokohama Municipal Citizen’s Hospital, Kanagawa, Japan; ^5^ Department of Gastroenterology and Hepatology, Saitama City Hospital, Saitama, Japan; ^6^ Division of Gastroenterology, Department of Internal Medicine, National Hospital Organization Saitama National Hospital, Saitama, Japan; ^7^ Department of Gastroenterology and Hepatology, National Hospital Organization Tokyo Medical Center, Tokyo, Japan; ^8^ Department of Gastroenterology and Hepatology, Sano Kosei General Hospital, Tochigi, Japan; ^9^ Department of Gastroenterology and Hepatology, Hino Municipal Hospital, Tokyo, Japan; ^10^ Department of Gastroenterology, International University of Health and Welfare, School of Medicine, Chiba, Japan; ^11^ Department of Radiology, Keio University School of Medicine, Tokyo, Japan; ^12^ Department of Surgery, Keio University School of Medicine, Tokyo, Japan

**Keywords:** hepatocellular carcinoma, immune checkpoint inhibitor, atezolizumab plusbevacizumab, treatment sequence, treatment beyond disease progression

## Abstract

**Background:**

The clinical significance of treatment beyond progression (TBP) with immune checkpoint inhibitor-based therapy in hepatocellular carcinoma (HCC) remains unclear. As atezolizumab plus bevacizumab has become a first-line therapy for advanced HCC, understanding the real-world outcomes of TBP is increasingly relevant.

**Methods:**

We conducted a multicenter retrospective observational study involving 122 patients with unresectable HCC treated with atezolizumab plus bevacizumab across nine liver centers in Japan. Among patients who experienced radiologic progressive disease (PD), clinical outcomes were compared between those who continued treatment beyond progression (TBP group) and those who discontinued therapy. Overall survival (OS), tumor response, and subgroup analyses based on major vessel involvement (MVI) were evaluated.

**Results:**

Among patients with PD, the median OS was not reached in the TBP group, compared to 13.6 months in the non-TBP group (HR 2.04; 95% CI 1.02–4.07; p=0.0435). When stratified by MVI status, patients without MVI who received TBP had significantly longer OS (median not reached) than those who received palliative care (median 6.2 months; HR 11.2; 95% CI 3.89–32.5; p<0.001). Among patients with MVI, TBP did not confer an OS benefit over palliative care (median OS: 10.4 months *vs*. 7.4 months; HR 2.24; p=0.260), whereas switching to subsequent chemotherapy showed improved OS (median 23.1 months *vs*. 7.4 months; HR 7.33; 95% CI 1.44–37.3; p=0.0164). Multivariate analysis identified MVI as an independent negative prognostic factor (HR 5.17; 95% CI 1.34–20.0; p=0.0172), even after adjusting for AFP ratio at progression.

**Conclusions:**

This multicenter study suggests that continuation of atezolizumab plus bevacizumab beyond radiologic progression may improve survival outcomes in selected patients with unresectable HCC, particularly those without major vessel involvement. These findings support the integration of TBP into personalized treatment strategies in advanced HCC.

## 1 Introduction

Hepatocellular carcinoma (HCC) remains a significant global health concern. A recent study reported that HCC was among the top three causes of cancer-related deaths in 46 countries and ranked within the top five in 90 countries as of 2020. Projections indicate that if current incidence and mortality rates persist, the annual number of new liver cancer cases could increase by over 55% by 2040, reaching approximately 1.4 million new diagnoses and an estimated 1.3 million deaths (1).

Based on the results of the IMbrave150 trial, the combination of atezolizumab and bevacizumab demonstrated superiority over sorafenib in patients with unresectable HCC, establishing it as a standard first-line therapy for advanced HCC (2). This marked a significant shift in the treatment landscape, positioning immune checkpoint inhibitors (ICIs) as a key component of systemic therapy for this malignancy.

To optimize the clinical application of ICI-based therapies, it is important to consider their unique response kinetics. Atypical response patterns, such as pseudoprogression (3, 4) delayed responses (5), which differ from the conventional patterns observed with cytotoxic chemotherapy or molecular-targeted therapy, have been increasingly recognized. Moreover, it has been reported that ICIs may induce immune-related responses that are not adequately captured by conventional evaluation methods such as RECIST 1.1, further supporting the rationale for treatment beyond progression (4, 6). These phenomena provide a rationale for continuing treatment beyond initial disease progression (PD), even in the absence of immediate radiographic improvement. As a result, the concept of treatment beyond progression (TBP) has been incorporated into the design of some clinical trials and is gaining traction in real-world clinical decision-making for selected patients (7). Accumulating evidence from multiple studies across different tumor types, including melanoma and non-small cell lung cancer (NSCLC), suggests that continued ICI treatment beyond PD may offer clinical benefit in certain cases (8–13). In HCC, the safety of TBP has been reported in a patient cohort, including nine cases treated with atezolizumab plus bevacizumab, suggesting that TBP may represent a viable treatment option (14). However, the efficacy of TBP remains controversial. For instance, a recent study in NSCLC reported no significant clinical advantage of ICI continuation after PD (15), highlighting the importance of a cautious and personalized approach to treatment. Therefore, the clinical value of TBP should be carefully evaluated, and more refined patient stratification is essential to identify those most likely to benefit from this approach (16).

Given the current evidence and ongoing debate regarding treatment beyond progression, the present study aimed to evaluate the clinical utility of this approach in patients with hepatocellular carcinoma who received atezolizumab plus bevacizumab. By employing a multicenter cohort design, we sought to assess the real-world safety and efficacy of this strategy and contribute to the growing body of evidence on the optimal management of advanced HCC.

## 2 Materials and methods

### 2.1 Study design and patients

This retrospective, multicenter observational cohort study enrolled consecutive 122 patients who were recruited from nine participating institutions across Japan (Keio University Hospital, Saiseikai Utsunomiya Hospital, Yokohama Municipal Citizen’s Hospital, Saitama City Hospital, Tokyo Dental College Ichikawa General Hospital, National Hospital Organization Saitama Hospital, National Hospital Organization Tokyo Medical Center, Sano Kosei General Hospital, and Hino Municipal Hospital) between October 2020 and October 2023. Eligible patients were those with unresectable HCC who received atezolizumab and bevacizumab. Imaging features of HCC, as outlined by the American Association for the Study of Liver Diseases criteria and the Liver Imaging Reporting and Data System (LI-RADS), include arterial phase hyperenhancement, lesion size, washout appearance, enhancing capsule appearance, and threshold growth observed on multiphase contrast-enhanced CT or MRI (17, 18). A prior history of receiving other systemic chemotherapy was allowed. Patients with major autoimmune diseases or other contraindications to immunotherapy were excluded.

The study protocol was approved by the Keio University School of Medicine Research Ethics Committee (approval number: No.20170202). This study was conducted in accordance with the ethical principles of the 1975 Declaration of Helsinki and the Ethical Guidelines for Medical and Health Research Involving Human Subjects issued by the Ministry of Education, Culture, Sports, Science and Technology and the Ministry of Health, Labor and Welfare of Japan.

### 2.2 Medical care

All individuals received atezolizumab (1200 mg) plus bevacizumab (15 mg/kg) intravenously every three weeks until radiologic or clinical progression, unacceptable toxicity, or withdrawal by the individual or physician decision. Tumor assessments were conducted using contrast-enhanced CT or MRI approximately every 6–9 weeks, according to institutional standards.

Tumor responses were evaluated using RECIST version 1.1 and modified RECIST (mRECIST) criteria. Adverse events were assessed and graded using the National Cancer Institute Common Terminology Criteria for Adverse Events (CTCAE), version 5.0.

### 2.3 Definition of treatment beyond progression

Individuals were categorized into two groups according to their post-progression management. The TBP group included those who continued atezolizumab plus bevacizumab beyond radiologic progression, receiving at least one additional cycle of treatment. In contrast, the non-TBP group consisted of individuals who discontinued the therapy upon confirmation of PD. The decision to continue treatment beyond PD was made at the discretion of the attending physician, provided that the individual did not exhibit rapid clinical deterioration or severe treatment-related toxicity.

### 2.4 Outcome assessment

The primary endpoint of this study was overall survival (OS), defined as the time from the initiation of atezolizumab plus bevacizumab treatment to death from any cause. Secondary endpoints included the objective response rate (ORR) and disease control rate (DCR), which were evaluated using both the RECIST 1.1 and the mRECIST criteria. Tumor response was assessed through contrast-enhanced CT or MRI performed at regular intervals, based on each institution’s clinical protocol. In addition to response evaluation, exploratory analyses were conducted to identify clinical and tumor-related prognostic factors associated with OS. Subgroup survival analyses were further performed according to the presence or absence of significant covariates identified in multivariate Cox regression models, including major vessel involvement.

### 2.5 Statistical analysis

Data were analyzed using SPSS version 30 (IBM Corp., Armonk, NY, USA) and are expressed as medians with interquartile ranges or as averages ± standard deviations, as appropriate. Graphs and linear correlations were constructed using Prism 10.0 (GraphPad Software, Inc., San Diego, CA, USA). Differences between the groups were assessed using the Student t-test. Survival curves were estimated using the Kaplan–Meier method, and differences between the groups were assessed using the log-rank test. Cox proportional hazard models were applied for multivariate analysis to identify independent prognostic factors.

## 3 Results

### 3.1 Patient characteristics

**Figure 1:**
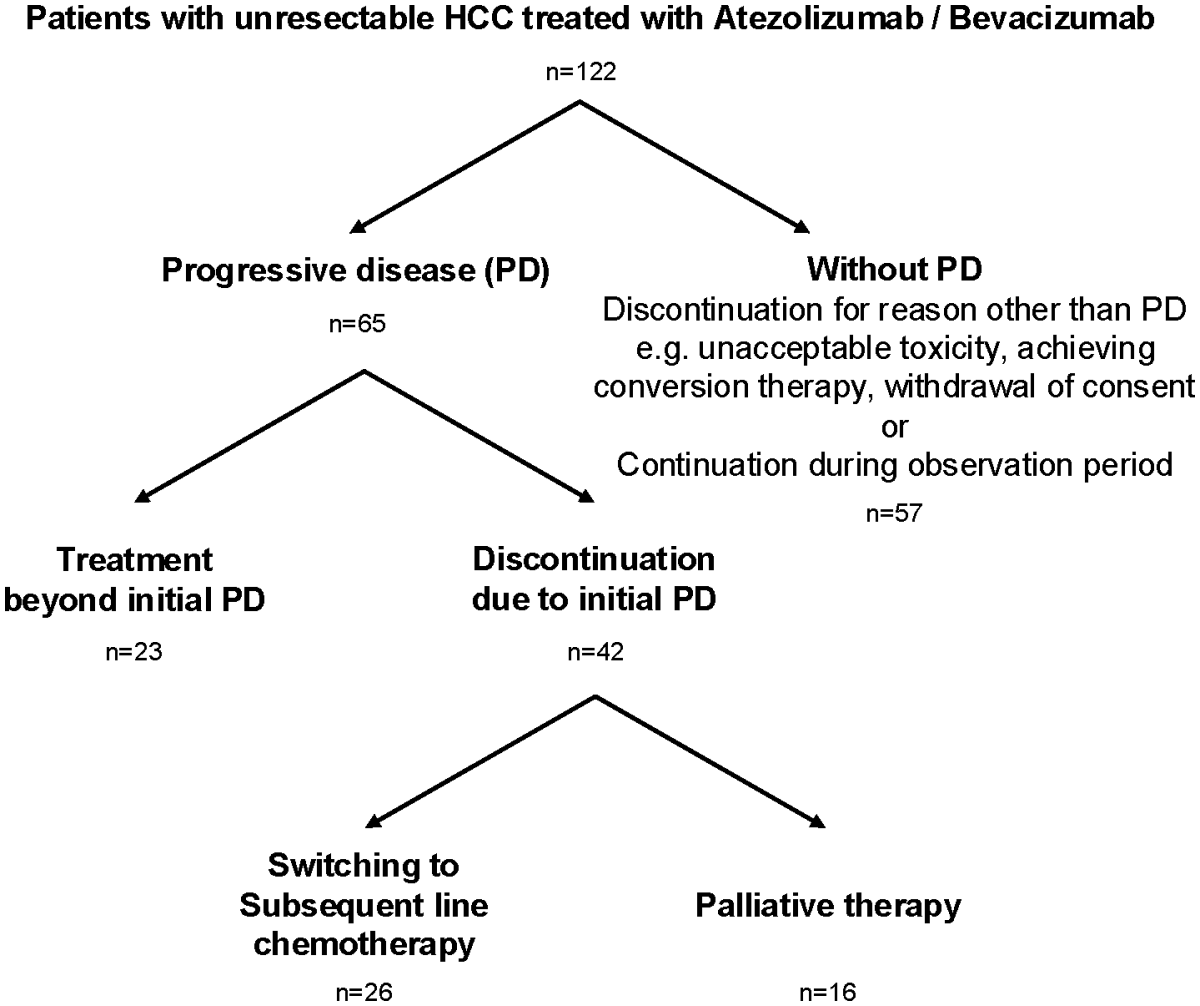
Flow chart of eligible patients.

A total of 122 individuals with unresectable HCC treated with atezolizumab plus bevacizumab were enrolled in nine liver centers across Japan between October 2020 and October 2023 (**Table 1**). The median age was 73 years (range 19–90 years), with 84% male. The underlying liver diseases were hepatitis C virus infection (29%), metabolic dysfunction-associated steatohepatitis/steatotic liver disease (MASH/MASLD; 27%), alcoholic liver disease (22%), and hepatitis B virus infection (15%). Child-Pugh A class was predominant (70%), and most individuals (66%) had an ECOG performance status of 0. Regarding tumor characteristics, 72% had Barcelona Clinic Liver Cancer (BCLC) stage C disease, 32% had major vessel involvement, and 40% had extrahepatic metastasis. Treatment with atezolizumab plus bevacizumab was administered as first-line therapy in 62% of individuals. Of the 122 individuals, 65 experienced radiologic progressive disease (PD). Among these, 23 individuals (35%) continued atezolizumab plus bevacizumab beyond the initial PD (TBP group), while 42 individuals (65%) discontinued treatment at the initial PD (non-TBP group). Of those who discontinued, 26 switched to subsequent-line chemotherapy, and 16 transitioned to palliative therapy (**Figure 1**). Comparison of baseline characteristics among TBP (n=23), subsequent chemotherapy (n=26), and palliative therapy (n=16) groups showed that initial line treatment was significantly more frequent in individuals receiving subsequent chemotherapy (73%) compared to the TBP group (39%, p=0.0156). Other baseline characteristics, including age, sex, ECOG performance status, Child-Pugh score, ALBI score, and tumor markers (AFP, DCP), did not significantly differ among the three groups (**Table 2**). As shown in **Supplementary Table 1**, patients who transitioned to palliative therapy after the first PD had significantly worse Child–Pugh scores, suggesting that impaired liver function was an important determinant of discontinuation. In contrast, no significant differences in liver function were observed between the TBP and subsequent therapy groups. However, patients in the subsequent therapy group were significantly more likely to have received atezolizumab plus bevacizumab as first-line therapy (**Table 2**).

**Table 1:** Characteristics of 122 patients.

Age (years)		AFP (ng/dl), n (%)	
median (range)	73 (19-90)	<200	81 (66)
≥60, n (%)	113 (93)	≥200	41 (34)
<60, n (%)	9 (7)	DCP (mAU/ml), n (%)	
Sex, n (%)		<400	57 (47)
Male	103 (84)	≥400	65 (53)
Female	19 (16)	BCLC stage, n (%)	
BMI (kg/m2) mean ± SD		B	34 (28)
mean ± SD	23.9 ± 3.8	C	88 (72)
Underlying liver disease, n (%)		Major vessel involvement, n (%)	
HCV	35 (29)	With	39 (32)
HBV	18 (15)	Without	83 (68)
Alcohol	27 (22)	Extrahepatic metastasis, n (%)	
MASH/MASLD	33 (27)	With	49 (40)
Others	9 (7)	Without	73 (60)
ECOG PS, n (%)		Metastatic sites, n (%)	
0	81 (66)	Lung	21 (17)
1	35 (29)	Lymph node	17 (11)
≥2	6 (5)	Bone	11 (9)
Child-Pugh class, n (%)		Peritoneum	12 (10)
A	85 (70)	Adrenal gland	3 (2)
B	37 (30)	Others	4 (3)
mALBI grade, n (%)		Line of treatment, n (%)	
1	27 (22)	1^st^ line	75 (62)
2a	36 (30)	2nd line	37 (30)
2b	53 (43)	3rd or subsequent line	10 (8)
3	6 (5)		

BMI, body mass index; HBV, hepatitis B virus; HCV, hepatitis C virus; MASH, metabolic dysfunction associated steatohepatitis; MASLD, metabolic dysfunction associated steatotic liver disease; ECOG PS, Eastern Cooperative Oncology Group Performance Status; mALBI grade, modified Albumin-Bilirubin grade; AFP, alpha-fetoprotein; DCP, des-γ-carboxy prothrombin; BCLC, The Barcelona Clinic Liver Cancer prognosis and treatment strategy.

**Table 2:** Patients characteristics of each group.

	Treatment beyond initial PD (reference)	Discontinuation due to 1st PD	Univariate	Switching to subsequent line chemotherapy	Univariate	Palliative therapy	Univariate
Variable	(N = 23)	(N = 42)	*p* value	(N = 26)	*p* value	(N = 16)	*p* value
Age (years) median (range)	69 (46-85)	71 (35-87)	0.353	71 (56-87)	0.262	71.5 (35-85)	0.663
Male, n (%)	21 (91)	36 (86)	0.502	24 (82)	0.898	12 (75)	0.168
BMI (kg/m2), mean ± SD	23.4 ± 2.91	24.6 ± 3.98	0.193	24.5 ± 4.10	0.271	24.8 ± 3.92	0.205
Hepatitis B, C virus infection, n (%)	9 (39)	13 (31)	0.507	9 (35)	0.744	4 (25)	0.353
ECOG PS, 0-1	21 (91)	41 (98)	0.259	26 (100)	0.0773	15 (94)	0.776
Child-Pugh score, mean ± SD	6.0 ± 1.1	6.0 ± 0.88	0.863	5.8 ± 0.86	0.510	6.4 ± 0.81	0.205
ALBI score, mean ± SD	-2.24 ± 0.505	-2.23 ± 0.522	0.900	-2.38 ± 0.500	0.325	-1.97 ± 0.461	0.0896
AFP (ng/dl), mean ± SD	937 ± 1654	7865 ± 23751	0.169	4069 ± 10803	0.176	14036 ± 35804	0.0863
DCP (mAU/ml), mean ± SD	3673 ± 9884	10188 ± 28552	0.295	8139 ± 16351	0.263	13391 ± 41571	0.286
BCLC stage B, n (%)	6 (26)	12 (29)	0.830	10 (38)	0.355	2 (13)	0.290
Major vessel involvement, n (%)	7 (30)	16 (38)	0.535	10 (38)	0.555	6 (38)	0.646
Extrahepatic metastasis, n (%)	13 (57)	17 (40)	0.215	8 (31)	0.0678	9 (56)	0.987
Initial line treatment, n (%)	9 (39)	28 (67)	0.0319*	19 (73)	0.0156*	9 (56)	0.291

Asterisks indicate statistically significant differences of means (0.01 ≤ *p < 0.05; 0.001 ≤ **p < 0.01; ***p < 0.001).

### 3.2 Radiological response and adverse event before first PD

The ORR and DCR in the TBP group, according to the mRECIST criteria, were 17% and 48%, respectively. In comparison, the subsequent chemotherapy group had ORR and DCR of 23% and 54%, respectively, and the palliative therapy group had ORR and DCR of 31% and 38%, respectively (**Table 3**). As shown in **Supplementary Table 2**, the frequency of grade ≥3 adverse events prior to the first PD did not significantly differ among the TBP, subsequent therapy, and palliative groups. Importantly, in the TBP group, we also identified six patients who were initially classified as PD but were later reclassified as SD, and one patient who was reclassified as PR upon subsequent evaluation.

**Table 3:** Best radiological responce of each group.

	Whole cohort N=122		Treatment beyond initial PD (reference) N=23		Switching to subsequent line N=26		Palliative therapy N=16	
Responce N (%)	mRECIST	RECIST	mRECIST	RECIST	mRECIST	RECIST	mRECIST	RECIST
CR	2 (2)	2 (2)	0 (0)	0 (0)	0 (0)	0 (0)	0 (0)	0 (0)
PR	36 (30)	28 (23)	4 (17)	3 (13)	6 (23)	4 (15)	5 (31)	3 (19)
SD	39 (32)	46 (38)	7 (30)	8 (35)	8 (31)	10 (38)	1 (6)	2 (13)
PD	34 (28)	35 (29)	12 (52)	12 (52)	12 (46)	12 (46)	10 (63)	11 (69)
ND	11 (9)	11 (9)	0 (0)	0 (0)	0 (0)	0 (0)	0 (0)	0 (0)
ORR	38 (34)	30 (27)	4 (17)	3 (13)	6 (23)	4 (15)	5 (31)	3 (19)
DCR	77 (69)	76 (68)	11 (48)	11 (48)	14 (54)	14 (54)	6 (38)	5 (31)

CR, complete response; PR, partial response; SD, stable disease; PD, progressive disease; ND, not determined; ORR, overall response rate; DCR, disease control rate.

### 3.3 Overall survival and progression-free survival

**Figure 2:**
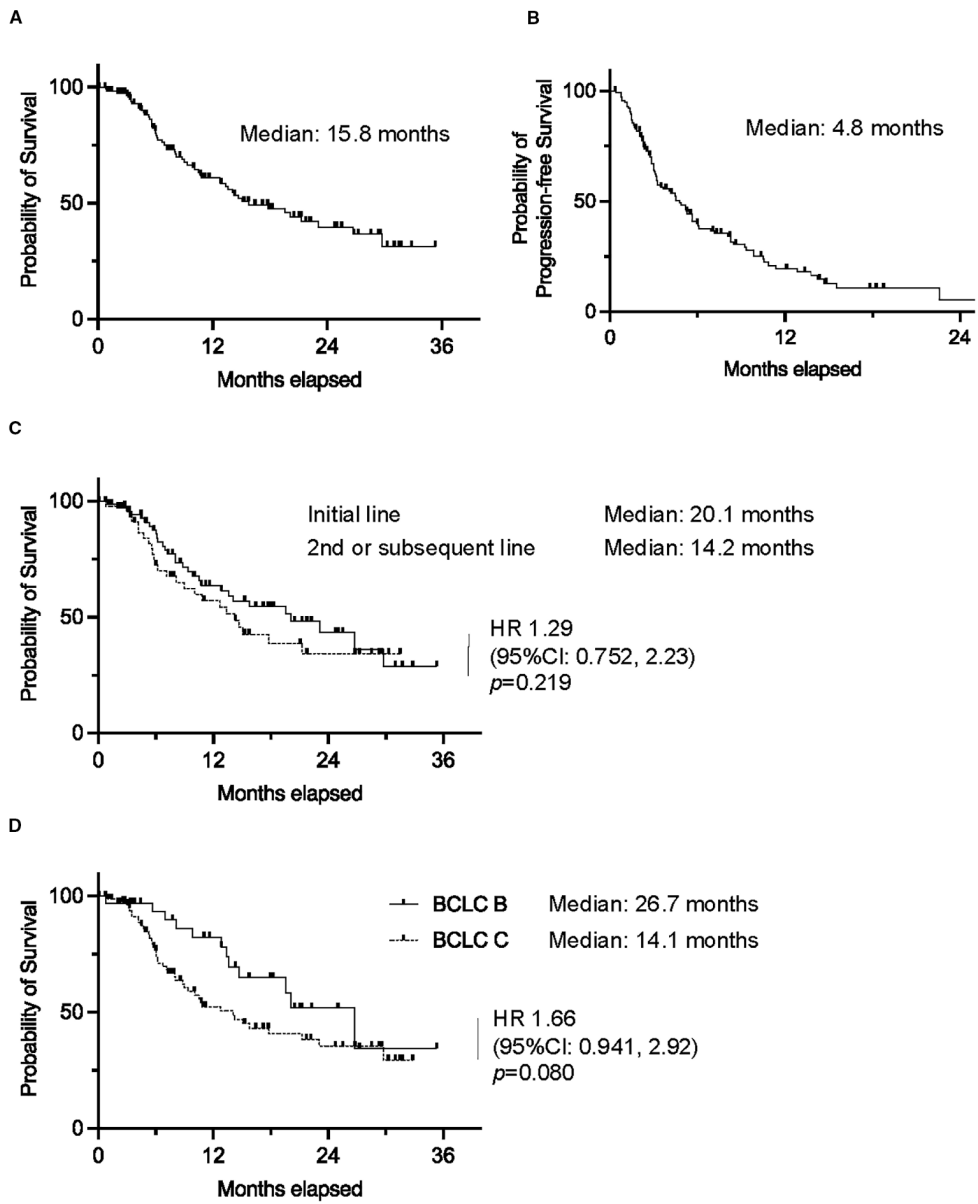
Kaplan–Meier curves of **(A)** overall survival and **(B)** progression-free survival in the whole cohort. Kaplan–Meier curves of overall survival in **(C)** patients who received atezolizumab plus bevacizumab as first-line or second/subsequent-line chemotherapy, and **(D)** patients with BCLC stage B or **(C)** (0.01 ≤ *p < 0.05).

The median OS of the entire cohort was 15.8 months, and the median progression-free survival (PFS) was 4.8 months. Individuals treated in the first-line setting demonstrated a longer median OS (20.1 months) compared to those treated in the second or subsequent lines (14.2 months), although this difference was not statistically significant (HR 1.29, 95% CI 0.752–2.23, p=0.219). Similarly, individuals with BCLC stage B disease tended to have longer OS (median 26.7 months) compared to those with BCLC stage C disease (median 14.1 months; HR 1.66, 95% CI 0.941–2.92, p=0.080) (**Figure 2**).

### 3.4 Survival outcomes by treatment strategy post-PD

**Figure 3:**
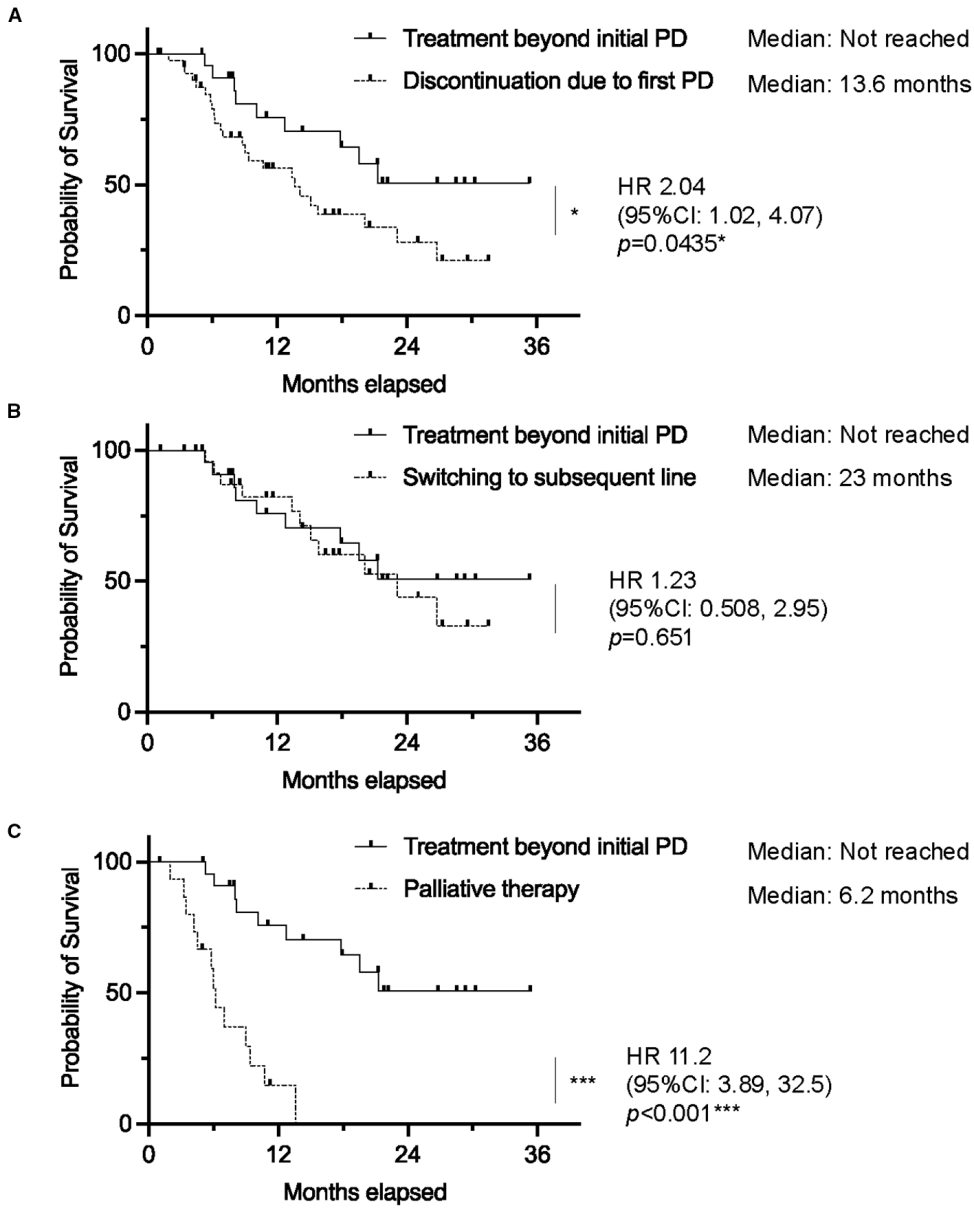
Kaplan–Meier curves of overall survival in patients who received atezolizumab plus bevacizumab, comparing **(A)** treatment beyond initial progressive disease (TBP) *vs*. discontinuation due to initial progressive disease (PD), **(B)** TBP *vs*. Switching to subsequent line, and **(C)** TBP *vs*. Palliative therapy. (0.01 ≤ *p < 0.05; 0.001 ≤ **p < 0.01; ***p < 0.001). *vs*: versus.

Median OS was not reached in the TBP group, significantly outperforming the non-TBP group (median OS: 13.6 months; HR 2.04, 95% CI 1.02–4.07, p=0.0435). Moreover, when stratified, individuals receiving TBP showed a significantly better OS compared to those receiving palliative therapy (HR 11.2, 95% CI 3.89–32.5, p<0.001), while no significant difference was observed between the TBP and subsequent chemotherapy groups (HR 1.23, 95% CI 0.508–2.95, p=0.651) (**Figure 3**).

### 3.5 Factors contributing to OS in TBP group

In univariate analysis, major vessel involvement (MVI) significantly worsened survival outcomes (HR 5.60, 95% CI 1.47–21.3, p=0.0115). This remained significant in multivariate analysis (HR 5.17, 95% CI 1.34–20.0, p=0.0172). Other factors, including age, sex, BMI, hepatitis virus infection, ECOG performance status, BCLC stage, extrahepatic metastasis, initial line treatment, Child-Pugh score, ALBI score, AFP, and DCP, were not significantly associated with OS. Notably, MVI was also identified as an independent factor associated with OS in a multivariate analysis that included the AFP ratio (AFP at PD diagnosis/AFP at treatment initiation) as a covariate, which was considered to reflect early treatment response (**Table 4**).

**Table 4:** Factors contributed to overall survival in patients who continued atezolizumab with bevacizumab treatment beyond initial diagnosis of progressive disease.

	Unadjusted		Multivariate	
Variable	HR (95% CI)	*p* value	HR (95% CI)	*p* value
Age (years)	0.972 (0.912-1.04)	0.416		
Male	0.267 (0.0295-2.41)	0.240		
BMI (kg/m2)	0.851 (0.591-1.14)	0.309		
Hepatitis B, C virus infection	0.571 (0.141-2.31)	0.432		
ECOG PS 0-1	0.990 (0.123-7.94)	0.992		
BCLC stage B	1.49 (0.305-7.26)	0.622		
Major vessel involvement	5.60 (1.47-21.3)	0.0115*	5.17 (1.34-20.0)	0.0172*
Extrahepatic metastasis	1.28 (0.318-5.16)	0.727		
Initial line treatment	0.725 (0.180-2.92)	0.651		
At the initiation of treatment				
Child-Pugh score	1.38 (0.767-2.40)	0.261		
ALBI score	2.06 (0.450-10.1)	0.352		
AFP (ng/dl)	2.06 (0.263-10.7)	0.448		
DCP (mAU/ml)	1.16 (0.0396-7.91)	0.905		
At the diagnosis of 1st PD				
Child-Pugh score	1.20 (0.846-1.62)	0.273		
ALBI score	2.21 (0.875-5.17)	0.0895		
AFP (ng/dl)	1.63 (0.151-8.66)	0.633		
DCP (mAU/ml)	4.86 (0.439-33.6)	0.172		
AFP ratio (PD Diagnosis / Initiation of Tx)	1.50 (0.707-2.81)	0.262	1.39 (0.581-2.86)	0.425

Asterisks indicate statistically significant differences of means (0.01 ≤ *p < 0.05; 0.001 ≤ **p < 0.01; ***p < 0.001).

### 3.6 Impact of major vessel involvement

**Figure 4:**
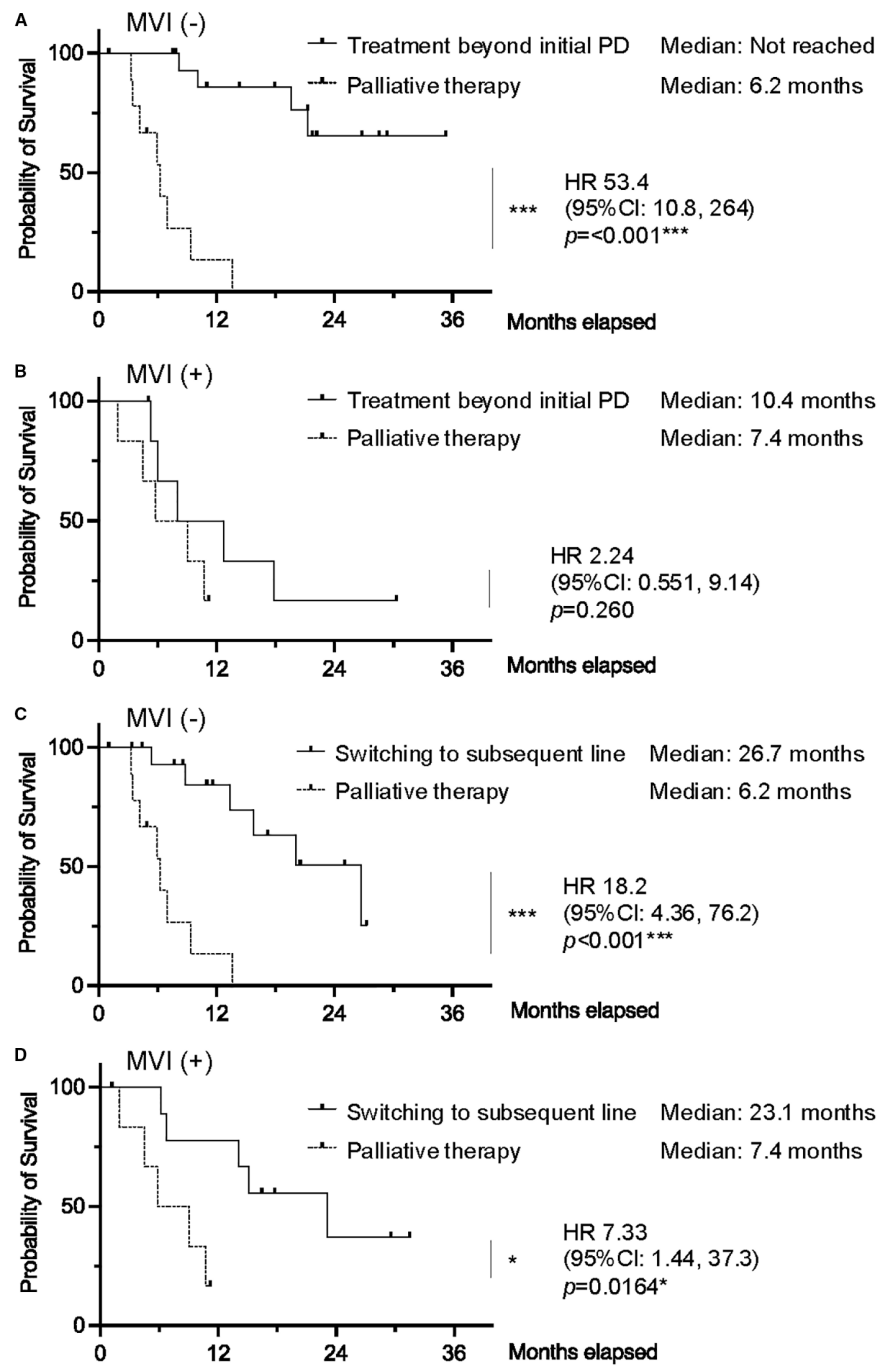
Kaplan–Meier curves of overall survival comparing TBP *vs*. palliative therapy in patients **(A)** without macrovascular invasion (MVI) and **(B)** with MVI. Kaplan–Meier curves of overall survival comparing switching to subsequent line *vs*. palliative therapy in patients **(C)** without MVI and **(D)** with MVI. (0.01 ≤ *p < 0.05; 0.001 ≤ **p < 0.01; ***p < 0.001). *vs*: versus.

Individuals without MVI who received TBP had significantly improved OS compared to those receiving palliative therapy (median OS not reached *vs*. 6.2 months; HR 53.4, 95% CI 10.8–264, p<0.001). Similarly, individuals without MVI who switched to subsequent chemotherapy also had significantly better OS compared to those receiving palliative therapy (median OS 26.7 months *vs*. 6.2 months; HR 18.2, 95% CI 4.36–76.2, p<0.001). Among individuals with MVI, differences in survival outcomes between TBP and palliative therapy groups were not statistically significant (HR 2.24, 95% CI 0.551–9.14, p=0.260). In this subgroup, although the survival benefit of switching to subsequent chemotherapy over palliative therapy was maintained (HR 7.33, 95% CI 1.44–37.3, p=0.0164), the superiority of TBP over palliative therapy was no longer observed (**Figure 4**).

## 4 Discussion

In this multicenter retrospective study, we demonstrated that TBP with atezolizumab plus bevacizumab was associated with significantly improved OS compared to immediate treatment discontinuation in individuals with unresectable HCC. Notably, individuals receiving TBP showed superior OS compared to those who transitioned directly to palliative care, highlighting the potential benefit of this strategy in carefully selected individuals. Additionally, OS did not significantly differ between the TBP group and those who transitioned to subsequent systemic therapy.

The combination therapy of atezolizumab and bevacizumab has demonstrated a favorable safety profile and tolerability in individuals with unresectable HCC, particularly when compared to other systemic therapies (7). In the phase III IMbrave150 trial, the incidence of treatment discontinuation due to adverse events was relatively low in the atezolizumab plus bevacizumab group compared with the sorafenib group. Furthermore, patient-reported outcomes from the same trial indicated that the combination therapy was associated with a longer median time to deterioration in quality of life (11.2 months *vs*. 3.6 months), suggesting better tolerability from the individual’s perspective (2). Specifically, patient-reported outcomes from the IMbrave150 trial demonstrated clinically meaningful benefits in quality of life, functioning, and symptom relief with atezolizumab plus bevacizumab compared to sorafenib, including significantly reduced risks of deterioration in appetite loss, diarrhea, fatigue, and pain. These improvements further underscore the potential for atezolizumab and bevacizumab to positively impact individuals’ overall quality of life and daily functioning during treatment (19).

The favorable safety profile is particularly important when considering treatment beyond progression, as prolonged therapy requires sustained tolerability. Moreover, the absence of a survival difference between the TBP group and those who transitioned to subsequent therapy is an important observation. Although the favorable safety profile of atezolizumab plus bevacizumab may have contributed to improved OS—particularly by reducing treatment-related interruptions or complications and thus potentially confounding the interpretation of the effects of continued treatment—these findings suggest that when OS is comparable, continuing atezolizumab plus bevacizumab may be a valuable strategy for preserving or improving quality of life (QOL). This approach may help individuals avoid the potential QOL decline associated with switching to other systemic therapies, further supporting the clinical rationale for TBP in appropriate individuals.

The comparable frequency of severe adverse events across groups suggests that the improved survival observed in the TBP group cannot simply be attributed to preferential inclusion of patients with lower toxicity profiles. Furthermore, the observation that a subset of patients in the TBP group (six initially classified as PD later reclassified as SD, and one as PR) experienced subsequent tumor control is consistent with pseudoprogression or delayed immune responses, phenomena characteristic of immunotherapy. These findings provide internal evidence supporting the biological plausibility of TBP as a valid treatment strategy in carefully selected cases.

The concept of TBP is increasingly recognized in oncology practice, particularly with ICIs, given their distinct response patterns, including pseudoprogression and delayed tumor responses. Previous studies in melanoma and NSCLC have also shown potential clinical benefits of continued ICI treatment beyond radiologic progression in selected cases (8–13). Our findings extend this observation to the treatment of advanced HCC, underscoring the importance of individual selection and careful clinical decision-making in managing treatment after radiologic progression.

Importantly, in our cohort, MVI was identified as a significant negative prognostic factor for OS in individuals undergoing TBP. This finding is consistent with previous studies that have repeatedly identified MVI as a marker of aggressive tumor behavior and a predictor of poor prognosis in advanced hepatocellular carcinoma (20, 21). Our multivariate analysis supported this observation by demonstrating that MVI independently predicted worse OS outcomes, even after adjusting for early treatment response as measured by the AFP ratio (AFP at progression diagnosis divided by AFP at baseline). Furthermore, subgroup analyses revealed differential treatment benefits based on the presence or absence of MVI. This may reflect the fact that individuals with MVI experience rapid disease progression and may not have sufficient time to benefit from the delayed therapeutic effects that TBP often requires, such as immune-mediated responses.

Furthermore, subgroup analyses revealed differential treatment benefits based on the presence or absence of MVI. Specifically, among individuals without MVI, TBP was associated with significantly improved survival compared to palliative therapy, whereas this benefit was not observed in individuals with MVI. In contrast, switching to subsequent chemotherapy maintained survival advantages over palliative care, even in MVI-positive individuals. These findings suggest that subsequent chemotherapy rather than TBP may be a more appropriate strategy for individuals with aggressive tumor features such as MVI, underscoring the necessity of individualized treatment strategies based on tumor biology and individual condition. Individuals with MVI generally have significantly poorer prognoses due to rapid disease progression and limited treatment responsiveness.

The lack of OS benefit from TBP in this subgroup may be attributed to their inability to wait for delayed treatment effects that TBP might require. Therefore, TBP should not be recommended for individuals with MVI, as the timeframe for realizing clinical benefit may be insufficient in this population. Because TBP often requires time to exert its therapeutic effects, particularly in cases where immune-related mechanisms are involved, individuals with MVI may not survive long enough to realize those benefits. Recent expert consensus has emphasized a shift away from rigid staging-based treatment algorithms toward more personalized treatment allocation strategies (22, 23) Moreover, there is emerging recognition that even in cases of slow progressive disease (slow PD), where tumors increase gradually without rapid clinical deterioration, continued therapy may still confer clinical benefit (24, 25). Our findings, particularly the utility of TBP in non-MVI individuals, support these personalized frameworks and call for treatment strategies that flexibly integrate individual and tumor characteristics into decision-making.

### 4.1 Limitations

Limitations of this study include its retrospective nature and potential selection bias inherent in observational studies. The decision for TBP was left to the discretion of the treating physician, which could introduce heterogeneity in individual management. Although additional analyses helped clarify some decision-making factors, including impaired liver function and availability of subsequent therapy, unmeasured confounders may remain. Moreover, despite being multicenter, the sample size in subgroup analyses was relatively limited, which may impact the statistical power and generalizability of the findings.

## 5 Conclusions

In conclusion, our study suggests that continuation of atezolizumab plus bevacizumab beyond radiologic progression may offer survival benefits for selected individuals with unresectable HCC, particularly those without major vessel involvement. In the absence of OS differences with subsequent therapy, TBP may provide added value by helping maintain or improve QOL. While this study focused on ICI therapy combined with VEGF inhibition, future investigations of pure ICI regimens—which represent one of the mainstays of HCC treatment—may offer an effective strategy for VEGF-intolerant patients. Importantly, this study highlights the potential of TBP as a treatment strategy in selected patients with advanced HCC. In the coming years, prospective trials and real-world data will be essential to determine whether TBP can be incorporated into treatment algorithms.

## Data Availability

The datasets presented in this article are not readily available because Research data are not publicly available on legal or ethical grounds. Further inquiries can be directed to the corresponding author. Requests to access the datasets should be directed to Nobuhito Taniki, nobuhitotaniki@keio.jp.
