# Supplementary material for: Continuation of atezolizumab plus bevacizumab beyond initial progressive disease: clinical benefits in patients with unresectable hepatocellular carcinoma – a multicenter cohort study
Source: Front Immunol. 2025 Sep 11;16:1653456. doi: 10.3389/fimmu.2025.1653456 (PMC12460182; doi:10.3389/fimmu.2025.1653456)
Supplement: Supplementary file 1 [file DataSheet1.pdf]

Supplementary Material

Continuation of atezolizumab plus bevacizumab beyond initial progressive disease: clinical benefits in patients with unresectable hepatocellular carcinoma – a multicenter cohort study

Takaya Tabuchi[1], Nobuhito Taniki[1*], Keisuke Ojiro[1,6], Ryosuke Kasuga[1], Yukie Nakadai[1], Po-Sung Chu[1], Shingo Usui[1], Shunsuke Shiba[2], Toshiyuki Tahara[2], Hirokazu Komatsu[3], Yuriko Fujita[3], Fumihiko Kaneko[4], Hitomi Hoshi[4], Akihiro Yamaguchi[5], Seiichiro Fukuhara[7], Yukishige Okamura[8], Hideaki Kanamori[9], Hirotoshi Ebinuma[10], Masashi Tamura[11], Jitsuro Tsukada[11], Yasushi Hasegawa[12], Yuta Abe[12], Minoru Kitago[12], Masahiro Jinzaki[11], Yuko Kitagawa[12], Takanori Kanai[1] and Nobuhiro Nakamoto[1*]

Supplementary Table 1. Baseline clinical characteristics at the time of first radiologic progression according to treatment group.

Variable	Treatment beyond initial PD (reference) (N=23)	Discontinuation due to 1st PD (N=42)	Univariate *p* value	Switching to subsequent line chemotherapy (N=26)	Univariate *p* value	Palliative therapy (N=16)	Univariate *p* value
Child-Pugh score, mean±SD	6.6±1.6	6.9±1.7	0.530	6.3±1.3	0.480	7.9±2.0	0.0444*
ALBI score, mean±SD	-1.98±0.618	-1.92±0.646	0.723	-2.00±0.679	0.908	-1.78±0.581	0.336

Asterisks indicate statistically significant differences of means ($0.01 \leq$ * $p < 0.05$; $0.001 \leq$ ** $p < 0.01$; *** $p < 0.001$).

Supplementary Table 2. Incidence of grade ≥3 adverse events before the first PD assessment across treatment groups

Adverse event, n (%)	Treatment beyond initial PD (N=23)		Switching to subsequent line chemotherapy (N=26)		Palliative therapy (N=16)		
	Grade3 n (%)	Grade 4/5 n (%)	Grade3 n (%)	Grade 4/5 n (%)	Grade3 n (%)	Grade 4/5 n (%)	P value
**Any event**	**6 (26)**	**0 (0)**	**4 (15)**	**0 (0)**	**2 (13)**	**0 (0)**	**0.4971**
Proteinuria	2 (9)	0 (0)	1 (4)	0 (0)	0 (0)	0 (0)	
Ascites	1 (4)	0 (0)	1 (4)	0 (0)	1 (6)	0 (0)	
Aspartate aminotransferase increase	0 (0)	0 (0)	1 (4)	0 (0)	1 (6)	0 (0)	
Alanine aminotransferase increase	0 (0)	0 (0)	1 (4)	0 (0)	1 (6)	0 (0)	
Hypertension	0 (0)	0 (0)	0 (0)	0 (0)	1 (6)	0 (0)	
Rash	0 (0)	0 (0)	1 (4)	0 (0)	0 (0)	0 (0)	
Decrease appetite	1 (4)	0 (0)	0 (0)	0 (0)	0 (0)	0 (0)	
Diarrhea	1 (4)	0 (0)	0 (0)	0 (0)	0 (0)	0 (0)	
Pyrexia	0 (0)	0 (0)	1 (4)	0 (0)	0 (0)	0 (0)	
Anemia	1 (4)	0 (0)	0 (0)	0 (0)	0 (0)	0 (0)	
Intestinal obstruction	1 (4)	0 (0)	0 (0)	0 (0)	0 (0)	0 (0)	
Peritoneal infection	1 (4)	0 (0)	0 (0)	0 (0)	0 (0)	0 (0)	
